# Establishment of ultrasound-guided stellate ganglion block in rats

**DOI:** 10.3389/fnins.2022.1061767

**Published:** 2023-01-12

**Authors:** Shi-zhu Lin, Lu Chen, Yi-jie Tang, Cheng-jie Zheng, Peng Ke, Meng-nan Chen, Hai-xing Wu, Yu Chen, Liang-cheng Qiu, Xiao-dan Wu, Kai Zeng

**Affiliations:** ^1^Department of Anesthesiology, Anesthesiology Research Institute, The First Affiliated Hospital of Fujian Medical University, National Regional Medical Center, Binhai Campus of the First Affiliated Hospital, Fujian Medical University, Fuzhou, China; ^2^Department of Anesthesiology, Fujian Provincial Hospital, Fujian Provincial Clinical Medical College, Fujian Medical University, Fuzhou, Fujian, China

**Keywords:** ultrasound-guided, blind detection, rat model, protocol, stellate ganglion block (SGB)

## Abstract

**Background:**

A novel protocol for accurate stellate ganglion block under ultrasound guidance was designed in rats. This technique raises the success rate of stellate ganglion block and reduces the incidence of brachial plexus and vagus nerve block.

**Methods:**

Fifty-six Sprague-Dawley were randomly divided into an ultrasound-guided group (*n* = 28) and a blind technique group (*n* = 28). The rats in the blind technique group were injected with 1.5% lidocaine mixed with methylene blue after signs of brachial plexus stimulation were elicited. The lateral side of the cephalic brachial vein was located under the first rib, where lidocaine was injected into the rats in the ultrasound-guided group. The up-and-down sequential method of Dixon was used to determine the minimum effective volume for stellate ganglion block in rats. Furthermore, we calculated the required operative duration of the two methods and observed the difference in the lidocaine diffusion range between the two groups.

**Results:**

The minimum effective volume for stellate ganglion block in the ultrasound-guided group was 0.040 ml, and the 95% CI was 0.026–0.052 ml. In the blind technique group, the minimum effective volume was 0.639 ml, and the 95% CI was 0.490–0.733 ml. Within the 95% CI of the lowest effective volume, the incidence of brachial plexus block as a complication of stellate ganglion block under ultrasound guidance was 10.00%.

**Conclusion:**

Stellate ganglion block under ultrasound guidance is more accurate than blind detection, which the incidence of complications of stellate ganglion block under ultrasound guidance was significantly lower than under blind detection; the rate of methylene blue staining in the vagus nerve was significantly lower under ultrasound guidance.

## 1. Introduction

Stellate ganglion block (SGB) is a method with a broad range of applications for the prevention and treatment of chronic diseases, including the treatment of pain (Gunduz and Kenis-Coskun, 2017; Ueshima, 2019), the treatment of sleep disturbance after breast cancer (Jin et al., 2018), and complementary therapy between chronic ulcerative colitis and hemodialysis (Gunduz and Kenis-Coskun, 2017; Jin et al., 2018). An efficient and stable animal model is particularly important for the further study of SGB. The anatomical structure and function of the stellate nerve are similar in rats and humans (Abdi and Yang, 2005). The stellate ganglion, located at the level of the 2nd thoracic vertebra, is formed by the fusion of the inferior cervical ganglion and the first three thoracic sympathetic ganglia (C6-T3). Due to the opposite relationship of the dominant components, vagal blockade may cause failure of the expected therapeutic effect of SGB to meet expectations. The therapeutic mechanism is mainly associated with sympathetic nerve block (Filippiadis and Kelekis, 2016). The anatomical structure of the target area can be clearly revealed by ultrasound, and the needle, the catheter and drug diffusion can also be shown simultaneously. Therefore, ultrasound effectively increases the success rate of target nerve block and reduces complications (Liu et al., 2016; Kanna et al., 2019a). Ultrasound guided stellate ganglion block has been widely validated in many clinical trials of human subjects (Shibata et al., 2007; Gofeld et al., 2009; Aleanakian et al., 2020). In clinical trials, it has been confirmed that ultrasound guided stellate ganglion block can greatly reduce adverse reactions and complications related to blind injection, such as intravascular injection, hematoma formation, temporary paralysis of recurrent laryngeal nerve and esophageal injury (Lee et al., 2012; Narouze, 2012). However, ultrasound-guided SGB in rats has not yet been reported. This study aims to establish the methodology of ultrasound-guided rat SGB and we propose a clearly stated hypothesis that ultrasound-guided rat SGB is significantly better than traditional blind SGB. A new murine model for ultrasound-guided SGB was established in this study. This study provides a reference for the accurate preparation of animal models of SGB.

## 2. Materials and methods

### 2.1. Ethics

Our experiment has been approved by Animal Experimentation Committee of Fujian Medical University (No: FJMU IACUC 2020-0063).

### 2.2. Animals

Male Sprague-Dawley (SD) rats, 6–8 weeks old, weighing 220–260 g, were purchased from the experimental animal center of Fujian Medical University with the license number scxk 2016-0002. The animals were housed in separate cages and fed freely at the experimental animal center of Fujian Medical University. Rats were fasted for 12 h before SGB. The experiments were carried out in strict accordance with the relevant provisions of the experimental animal ethics committee of Fujian Medical University. Fifty-six rats were divided into the ultrasound-guided positioning group (*n* = 28) and the blind detection positioning group (*n* = 28).

### 2.3. Surgical procedures

**FIGURE 1:**
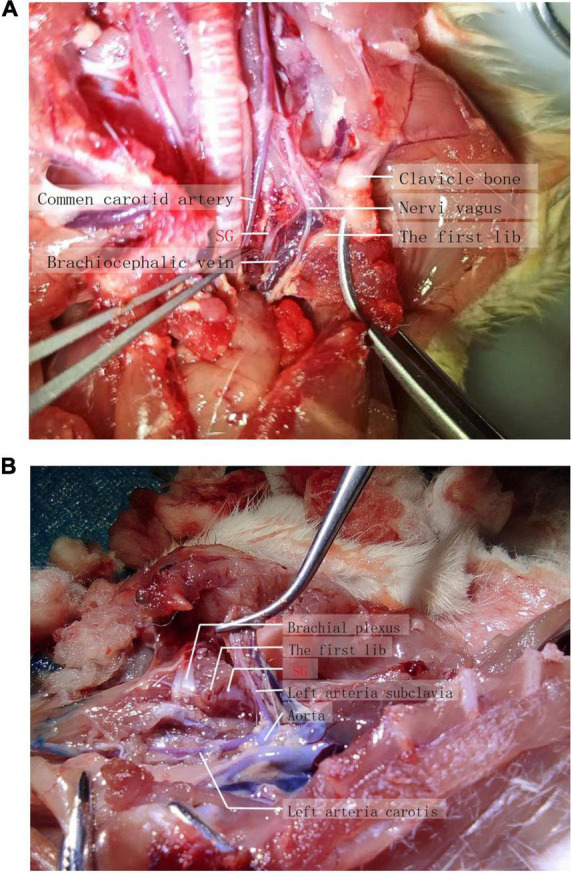
**(A,B)** Exact anatomical location of the stellate ganglion.

Rats were dissected to explore the location and distribution of the stellate ganglion. After anesthetization with 5% sevoflurane, the rats were sacrificed by injecting 2 ml of air into the tail vein. To expose the vagal sympathetic chain and the bifurcation of the carotid artery, the skin and fascia were separated layer by layer, and the sternocleidomastoid muscle and sternohyoid muscle were stripped. The superior cervical ganglion was separated after the bifurcation of the carotid artery. It was thus clear that the sympathetic nerve was adjacent to the superior cervical ganglion, where the vagus nerve and sympathetic nerve in the vagal sympathetic chain are separated. Stellate ganglia are distributed near the heart of the sympathetic nerve (Figures 1A, B). Observers and experimentalists should be blinded to the intervention and the effectiveness of blinding assessed to prevent systematic bias.

### 2.4. Blind localization for SGB

Rats were anesthetized with 5% sevoflurane in a mixture of 50% oxygen and air, and 2% sevoflurane was used to maintain anesthesia in rats. The sternoclavicular joint was used as the body surface marker for puncture. A no. 7 syringe was used to insert the needle vertically 0.3 cm outside the sternoclavicular joint (close to the lower edge of the clavicle during puncture). After the needle tip touched the brachial plexus nerve, the rats showed upper limb vibration on the corresponding side, indicating that the puncture was successful (D’Antoni, 2016). Lidocaine (1.5%) mixed with methylene blue was injected after the syringe tube was drawn back, without blood. After the injection, the rats were returned to their cages for observation. If the rats developed Horner syndrome within 5 min, the SGB was considered successful. The volume of lidocaine was investigated by the sequential method. According to the literature we reviewed and our pre-experiment (Kau et al., 2006; Li et al., 2022), the initial volume was set as 0.8 ml; if the block was successful, the volume in the next animal was reduced by 0.1 ml; if the block was not successful, the volume in the next animal was increased by 0.1 ml.

### 2.5. Ultrasound-guided SGB

**FIGURE 2:**
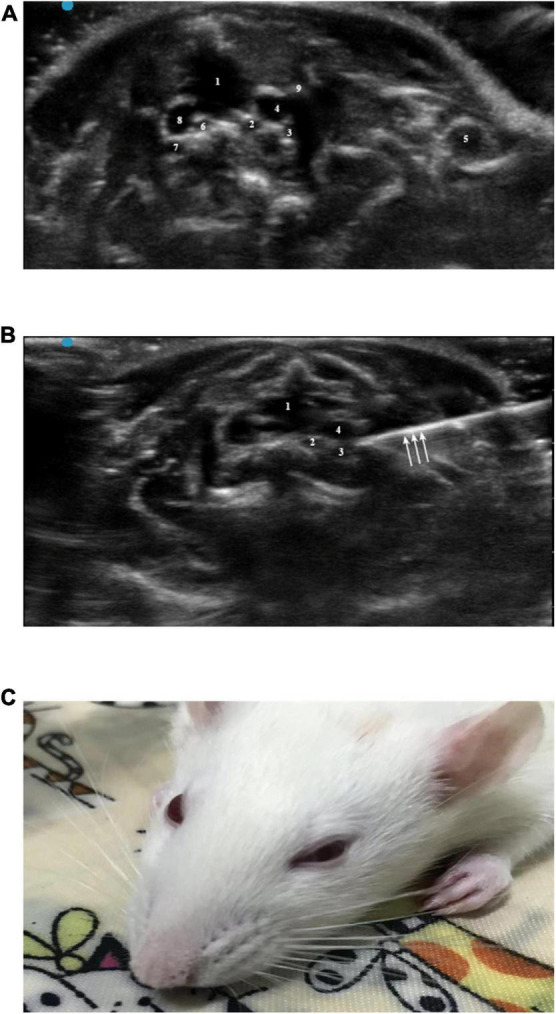
**(A)** Horizontal ultrasonography of the first intercostal space: 1, trachea; 2, left common carotid artery; 3, left subclavian artery; 4, left brachial vein; 5, left subclavian vein; 6, right common carotid artery; 7, right subclavian artery; 8, right brachial vein; 9, left first rib. **(B)** Ultrasound-guided SGB: 1, trachea; 2, left common carotid artery; 3, left subclavian artery; 4, left cephalic brachial vein; arrow, needle. **(C)** Successful ultrasound-guided left SGB in rats (Horner syndrome).

Sevoflurane (5%) was used to induce anesthesia, and the rats were placed supine on the operating table; 2% sevoflurane was used to maintain anesthesia. The skin around the clavicle (4 cm × 4 cm) was depilated with depilation cream. The 5- to 13-Hz probe of the Sonography Edge II system was placed in the left third to fourth intercostal space to locate the level of the rat atrium. Then, the probe was translated to the head of the rat to find the cephalic brachial vein, which was located in front of the common carotid artery and subclavian artery. Movement of the probe was continued to determine the position of the first rib. The block target area was located behind the brachiocephalic vein below the first rib (Figures 2A, B). Blood vessels were avoided during the needle insertion process. After the needle tip reached the target area, the syringe cylinder was drawn back to confirm that there was no blood, and then 1.5% lidocaine mixed with methylene blue was injected. After successful injection, the needle was withdrawn, and the puncture site was pressed. If signs of Horner syndrome were observed within 5 min after the rat recovered from anesthesia, SGB was considered successful. As mentioned above, the volume of lidocaine was investigated by the sequential method (Figure 2C). Since there are few literatures on ultrasound guided stellate ganglion block in rats, its initial volume is set according to our pre-experiment.

Sensory blockade (nociception assessment) and motor blockade (grasping and straightening tests) are used to evaluate whether there is brachial plexus block in rats after lidocaine injection (Zhang et al., 2019). The most intuitive is to observe the twitch of forelimbs and forepaws of rats. The number of rats in which SGB was successful and the time required for the block operation (from needle insertion to the end of injection) were recorded, and complications, such as brachial plexus block, hematoma, respiratory distress and death, were recorded.

### 2.6. Statistical analysis

SPSS 17.0 was used to process the data. According to the stop rule of the up-and-down sequential method of Dixon (Dixon, 1991; Pace and Stylianou, 2007), the sample size is determined by the simulation results. This study determines the sample size of 28 rats in each group. Quantitative data are expressed as the mean ± standard deviation (±s) and were statistically analyzed by independent samples *t*-tests. The qualitative data were analyzed by the chi square test or Fisher’s exact probability method. The lowest effective volume of lidocaine was calculated by the probit probability regression method and the up-and-down sequential method of Dixon, and the 95% confidence interval (95% CI) was calculated. *P* < 0.05 was considered to indicate a significant difference.

## 3. Results

### 3.1. Distribution range of lidocaine

In order to find out the exact anatomical range of lidocaine diffusion in ultrasound guided stellate ganglion block, we mixed 1.5% lidocaine with methylene blue, performed ultrasound guided stellate ganglion block, and killed the rats after Horner’s sign. Anatomically, it was found that the stellate ganglion was light blue when stained with methylene blue, while the brachial plexus remained the original gray white of nerve tissue (Figure 1B).

### 3.2. Anatomical characteristics of the stellate ganglion

**FIGURE 3:**
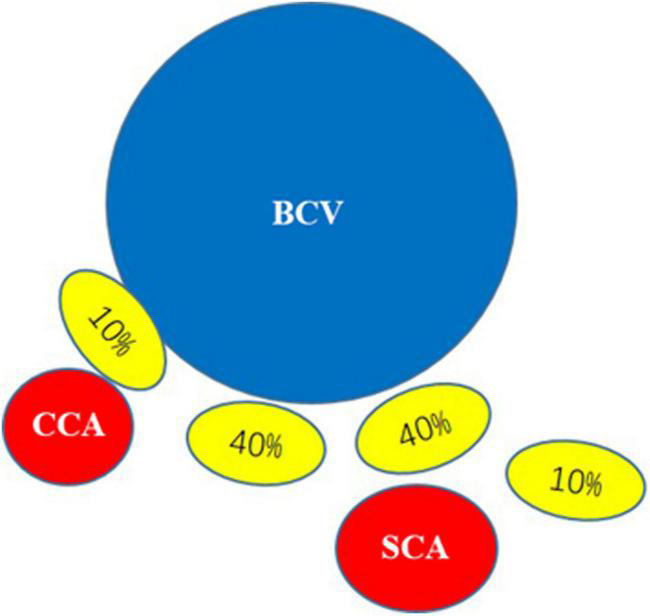
Positional relationship between the stellate ganglion and brachiocephalic vein: BCV, brachiocephalic vein; CCA, common carotid artery; SCA, subclavian artery. The yellow ellipse in the figure represents the position of the stellate ganglion, and the percentage of the yellow ellipse represents that the stellate ganglion is mostly located behind the first costal inferior brachiocephalic vein (40%), and can also be located behind the medial side (10%) or post-erolateral side (10%).

According to the local anatomy of the rat neck, the left stellate ganglion was mostly commonly located behind the brachiocephalic vein under the first rib but could also be located posterior medial or posterior lateral. It had a yellow-white, irregular star shape and multiple branches to the brachial plexus, head and face (Figure 3).

**FIGURE 4:**
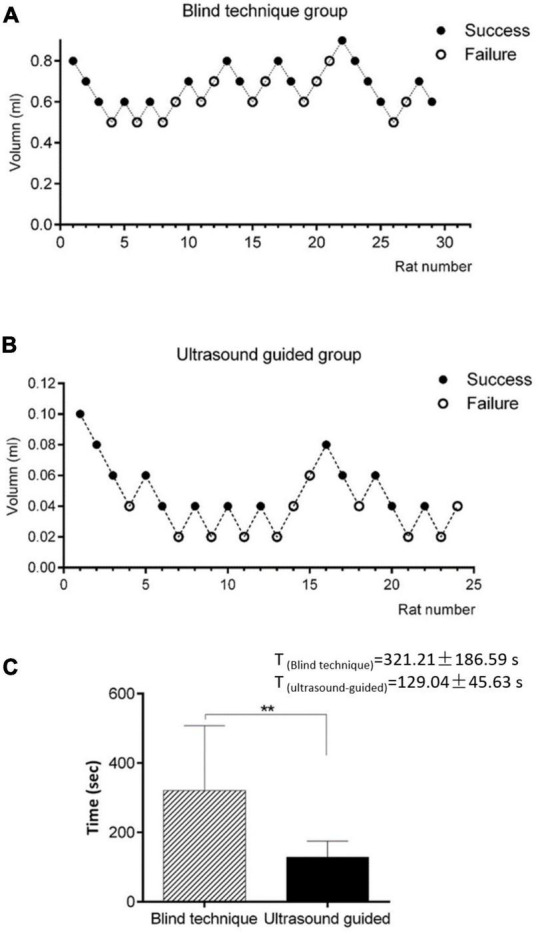
**(A)** Minimum effective volume for SGB in the blind technique group. The minimum effective volume for SGB in the ultrasound-guided group was 0.040 ml, the 95% CI was 0.026–0.052 ml. **(B)** Minimum effective volume for SGB in the ultrasound-guided group. The minimally effective volume for SGB in the blind technique group was 0.639 ml, and the 95% CI was 0.490–0.733 ml. **(C)** Comparison of the average operative duration between the two groups. Compared with blind technique, **P < 0.001. The average operative duration of ultrasound-guided SGB was significantly shorter than that of blind detection.

According to the up-and-down sequential method of Dixon, the minimum effective volume for the 1.5% lidocaine blind technique/ultrasound-guided SGB and 95% CI were calculated (Figures 4A, B). The differences were statistically significant. The minimum effective volume for SGB in the ultrasound-guided group was 0.040 ml, the 95% CI was 0.026–0.052 ml, while the minimally effective volume for SGB in the blind technique group was 0.639 ml, and the 95% CI was 0.490–0.733 ml.

### 3.3. Operative duration

The average operative duration of 28 ultrasound-guided SGB procedures was 129.04 ± 45.63 s. The average operative duration of 29 blind detection SGB procedures was 321.21 ± 186.59 s (Figure 4C). The difference was statistically significant (*P* < 0.001).

### 3.4. Incidence of brachial plexus block

On the premise of the same effect of SGB, observe the incidence of brachial plexus block as a complication by ultrasound-guided SGB, and the rats injected with lidocaine within the 95% CI of the lowest effective volume were selected (Table 1). For rats with lidocaine injection volumes of 0.026–0.052 ml, the estimated incidence of brachial plexus block in ultrasound-guided SGB was 10.00%.

**TABLE 1:** Incidence rate of brachial plexus block with lidocaine injection volume within 95% CI of the lowest effective volume.

	Brachial plexus blocked		Incidence of brachial plexus block
	Occur	Miss	
Ultrasound-guided group	1	9	10.00%

### 3.5. Other complications

There was no significant difference in the incidence of hemothorax, dyspnea, pneumothorax or death between the two methods (Table 2).

**TABLE 2:** The incidence of blood chest, dyspnea, pneumothorax and death in rats with stellate ganglion block under blind technique and ultrasound-guided group.

	Hematoma	Asphyxia	Pneumothorax	Death
Ultrasound guided group	4.16%	0%	0%	0%
Blind technique group	17.24%	13.79%	6.90%	6.90%

## 4. Discussion

This study initiated a new method for SGB in rats and verified its accuracy. Under ultrasound guidance, the operative duration, minimum effective volume of local anesthetic, and incidence of brachial plexus block, vagal block, respiratory depression and death were significantly reduced. These results confirm the reliability and safety of this technique.

Ultrasound-guided SGB or sympathetic trunk block in humans is generally performed in the neck (Baek et al., 2018; Singh et al., 2021). The human sympathetic trunk and the vagus nerve are located in different fascial layers (Yuan et al., 2017; Freitas et al., 2020). The long cervical muscle can be exhibited by ultrasound at the C6-7 level. Local anesthetic drugs could be injected on the surface to achieve SGB and perfectly evade the vagus nerve, achieving the effect of a simple sympathetic block. A clinical retrospective case study found that ultrasound guided stellate ganglion block was safe and effective in reducing sympathetic maintenance pain in patients with CRPS and neuropathic pain syndrome, and confirmed that the incidence of complications (3.9% hoarseness, 3.4% dysphagia, 0.6% hematoma) was lower than that of non-ultrasound guided techniques (Aleanakian et al., 2020). However, unlike humans, the rat neck sympathetic and vagus nerves are parallel in the same vascular sheath (Bookout and Gautron, 2021). Preliminary experimental results indicate that neck block probably blocks also the vagus nerve. Therefore, the first intercostal space was chosen as the target for SGB in this study. In this gap, the stellate ganglion is far from the vagus nerve and brachial plexus. Low-dose local anesthetics achieve a pure sympathetic nerve block and avoid brachial plexus block.

Traditional SGB in rats relies on body surface markers and the blind injection of drugs. Without sufficient experience, the probability of accidental injury of nerves or blood vessels is greatly increased, and the blockade effect is unsatisfactory (Filippiadis and Kelekis, 2016). The effect of traditional SGB will inevitably be affected by anatomical variations in rats, and an undesirable nerve blockade effect can be determined by the brachial plexus stimulation sign alone. Besides, various methods have been used to localize the stellate ganglion, such as CT (Christie and Martinez, 1995), MRI (Hogan and Erickson, 1992; Slappendel et al., 1995), and fluoroscopy (Elias, 2000; Abdi et al., 2004; Maki et al., 2016). Fluoroscopy-guided stellate ganglion block has been widely reported as a new technology (Maki et al., 2016). It shows the position of the needle and the diffusion range of drugs in real time through radiography, which is more suitable for providing diagnostic evaluation and has been proven effective in human clinical research (Elias, 2000). However, in animal experiments, fluoroscopy-guided stellate ganglion block needs to be carried out in a laboratory environment that can effectively resist radiation, such as a lead plate, which has high requirements for the laboratory, and also has the harm of radiation to laboratory personnel. Under the guidance of ultrasound, the stellate ganglion block of animals has no radiation, high repeatability, and can also visualize the blocking process and drug diffusion, so it is more feasible to use ultrasound to create models in animal experiments. Ultrasound technology clearly depicts the anatomical structures of muscles, intervertebral foramina, nerve roots, and blood vessels in the target area, thereby mediating navigation of the puncture needle to the pre-determined position to increase the nerve block success rate (Liu et al., 2016; Kanna et al., 2019b). In this study, the volume of drugs used was smaller and the operative duration was shorter in the ultrasound-guided group, indicating that the method is more stable and easier to master.

The anatomical exploration revealed that the brachial plexus ganglion was located at the upper edge of the first rib near the clavicle, while the stellate ganglion is located at the lower edge of the first rib. Brachial plexus irritation indicates the successful injection of drugs for SGB, which is contrary to the characteristics of the anatomical structure. It also reveals that the capacity required to achieve the effect of SGB is often larger than expected. The reason why this indication has been widely used may be the failure to find other positioning methods better than the blind probing method. Ultrasound guidance solves this problem and provides a better way to accurately establish the model.

Hemorrhage, nerve palsy, and pneumothorax are serious complications of SGB, which will affect the results of observation and research in experimental models (Okuda et al., 2016; Hirota et al., 2017). There were two cases of hematoma and two cases of pneumothorax in the blind detection group. This is attributed to the fact that the blind detection operation lacks sufficient precision in control of the needle tip and requires repeated puncture to induce brachial plexus irritation, which causes damage to blood vessels, pleura and nerves (Saranteas et al., 2019).

A systematic review of animal studies showed that the harmful effects of nerve block are mostly caused by mechanical damage, volumetric pressure and neurotoxicity (Sondekoppam and Tsui, 2017; Hewson et al., 2018; Barrington and Lirk, 2019). In this study, the minimum effective volume for rat SGB under ultrasound guidance was 0.020 ml, while the minimum effective volume for rat SGB under blind detection was 0.639 ml. Ultrasound guidance can effectively avoid the harmful effect of volumetric pressure and achieve better therapeutic effects with smaller volumes. Most nerve injuries are caused by mechanical stimulation (direct puncture trauma, pressure injury) or neurotoxic chemical factors (Sondekoppam and Tsui, 2017). The degree of nerve injury during nerve block depends on the angle and size of the puncture. Under visual conditions, the position of the needle, the angle of needle insertion and the diffusion of the drug are clearly displayed to pilot the puncture needle to the pre-determined position, minimizing the occurrence of mechanical injury (Gadsden et al., 2014). We found that within the 95% CI of the lowest effective volume, the complication rate of the ultrasound-guided method was significantly lower than that of the blind detection method. Methylene blue was mixed with 1.5% lidocaine to ascertain the exact range of lidocaine diffusion during ultrasound-guided SGB. The results confirmed that the lidocaine injected under ultrasound guidance was accurately distributed near the stellate ganglion without spreading to the brachial plexus. While ensuring the effect of SGB, brachial plexus block should be avoided as much as possible. In terms of the operative duration, the ultrasound-guided positioning method was significantly faster than the blind detection method, which has notable advantages. For the operator, it is easier to establish a rat SGB model under ultrasound guidance, and the model is more stable.

It is worth noting that there are still some shortcomings in this study. First, although ultrasound guidance technology was used, it could not provide an accurate image of the stellate ganglia. Thus, the method still depends on the positioning of the adjacent ganglia, blood vessels and bone tissues. This defect needs to be resolved by higher-resolution ultrasound. Second, the up-and-down sequential method of Dixon was used to investigate the minimum effective volume and ED95. However, this method is not sufficiently accurate to estimate the ED95. In further research, the partial coin sequential method will be used. Third, ultrasound-guided SGB surgery requires high-frequency ultrasound equipment; thus, this method is not as portable as the blind detection method. Fourth, due to the small number of experimental animals, it was difficult to compare the incidence of complications of SGB with ultrasound guidance and blind detection in terms of hemothorax and pneumothorax.

## 5. Conclusion

Ultrasound-guided SGB allowed visualization of the block process, and the effect of the block was definite. The SGB model established in this manner is more stable, avoiding complications to the greatest extent. Compared with traditional techniques, this method can be better applied in SGB-related research.

## Data availability statement

The raw data supporting the conclusions of this article will be made available by the authors, without undue reservation.

## Ethics statement

This animal study was reviewed and approved by Animal Experimentation Committee of Fujian Medical University (No: FJMU IACUC 2020-0063).

## Author contributions

KZ and X-DW: carried out the studies and modify the manuscript. S-ZL and LC: data collection, statistical analysis, repeated reading of the manuscript, and management of the author’s team. Y-JT, C-JZ, and PK: data collection, organization of data collection, and statistical analysis. M-NC: statistical analysis, methodological contributions, and repeated reading of the manuscript. H-XW and YC: data collection and discussion of the manuscript. L-CQ: repeated reading of the manuscript. All authors contributed to the article and approved the submitted version.
